# Evaluation of dual pathogen recognition receptor agonists as adjuvants for respiratory syncytial virus - virus-like particles for pulmonary delivery

**DOI:** 10.3389/fimmu.2025.1561297

**Published:** 2025-03-17

**Authors:** Ahmedali S. Mandviwala, Komal Liman, Anke L. W. Huckriede, Vidya A. Arankalle, Harshad P. Patil

**Affiliations:** ^1^ Department of Translational Virology, Interactive Research School for Health Affairs (IRSHA), Bharati Vidyapeeth (Deemed to be University), Pune, India; ^2^ Department of Medical Microbiology, University of Groningen, University Medical Center Groningen, Groningen, Netherlands

**Keywords:** respiratory syncytial virus (RSV), virus-like particles (VLPs), mucosal vaccine, pulmonary delivery, chimeric adjuvants

## Abstract

**Introduction:**

Respiratory syncytial virus (RSV) remains a significant global health concern, particularly for infants and young children in developing countries. Despite ongoing research efforts, an effective RSV vaccine has yet to be approved for widespread use. Use of two separate pattern recognition receptor (PRR) agonists as adjuvants in vaccine formulations has shown to enhance the immune response against the antigen. The limitation with the use of two adjuvants is that they need not necessarily bind to PRRs on the same cell. This study evaluates the efficacy of two different dual PRR binding chimeric molecules CL413 (TLR2/TLR7 agonist) and CL429 (TLR2/NOD2 agonist) as adjuvants for RSV virus-like particles (VLPs) delivered via the pulmonary route in mice for induction of mucosal and systemic immunity.

**Methods:**

BALB/c mice were immunized twice with the RSV-VLPs alone or adjuvanted with CL413, CL429, mixture of single PRR agonists Pam3CSK4+ L18-MDP or Pam3CSK4+ imiquimod via the pulmonary route. The mixture of single PRR agonists adjuvants was used as control for chimeric adjuvants. Immune responses were evaluated by measuring antibody levels in sera and respiratory tract; cytokine production, B and T cell responses in the lungs and spleen.

**Results:**

Pulmonary immunization with CL413-adjuvanted VLPs induced robust nasal IgA responses against the RSV F and G proteins, which was not observed for the other adjuvant combinations. CL413 also enhanced serum IgG levels and promoted a balanced Th1/Th2 response, as evidenced by IgG2a/IgG1 ratios. CL413 elicited strong pro-inflammatory responses in the lungs of mice, including elevated levels of IFN-γ, TNF-α, IL-6, and IL-17A. Flow cytometry analysis revealed increased numbers of tissue-resident class-switched B cells in the lungs of mice that were immunized with VLPs adjuvanted with CL413 and CL429. CD4+ and CD8+ T cell responses were also enhanced in both lungs and spleens of mice receiving VLPs adjuvanted with chimeric molecules to various extents. Mice immunized with formalin inactivated RSV (FI-RSV), which are used as the positive control for vaccine induced pathology after RSV challenge developed alveolitis, perivascular infiltration. While all the mice receiving adjuvanted VLP formulations showed protection against lung pathology after RSV challenge.

**Discussion:**

The lack of pathology, combined with the robust mucosal and systemic immune responses, suggests that pulmonary delivery of adjuvanted RSV-VLPs may provide effective protection without the risk of vaccine-enhanced disease. The study also demonstrates that the chimeric TLR2/TLR7 agonist CL413 is a promising adjuvant for RSV-VLPs to induce mucosal and systemic immune response and warrant further investigations in more advanced preclinical models.

## 1 Introduction

Respiratory Syncytial Virus (RSV) is a significant viral pathogen responsible for acute respiratory infections, particularly in infants and the elderly (1). First identified in the 1950s, RSV has since been recognized as a leading cause of bronchiolitis and pneumonia in children under five years of age, with an estimated global incidence exceeding 30 million cases annually (2). The virus poses a substantial public health burden, leading to millions of hospitalizations and considerable healthcare costs each year (3). Approximately 97% of global deaths due to RSV occur in low- and middle-income countries (LMICs) (4). The virus predominantly spreads through respiratory droplets when an infected person coughs or sneezes, which then infect the upper respiratory tract (URT) via nasopharyngeal or ocular mucosa (5, 6).

Pre-existing secretory IgA in the nasal cavity plays a crucial role in preventing the binding of the virus to its cellular receptors and further spreading of the virus to the lower respiratory tract (7). Immunization via the intramuscular route does not induce mucosal IgA and may not protect at the portal of entry. Therefore, targeting the respiratory tract, the primary site of RSV infection, may facilitate a more localized immune response and can lead to protection in the lungs (8).

Previously, we have shown that pulmonary and nasal delivery of the RSV-VLPs made employing prefusogenic-F (preFg) protein, glycoprotein (G) and matrix (M) proteins is equally effective for eliciting serum IgG, cellular response, and protection (9). The preFg form of RSV-fusion (F) protein as developed by Patel et al. demonstrates equal immunogenicity to the prefusion (preF) form, and effectively elicits antibodies targeting the most potent antigenic sites on the fusion protein, specifically sites Ø and V (10, 11). However, administration of these VLPs via either of these delivery routes did not induce mucosal antibodies, especially IgA. Additionally, the amount of systemic IgG2a antibodies, the protective antibody subtype in mice were low (9). An approach to enhance the IgA and IgG2a response is the addition of an adjuvant to the VLPs (12, 13).

An important class of adjuvants are the pathogen-recognition-receptors (PRRs) ligands. PRR ligands are pathogen-associated molecular patterns (PAMPs) that upon binding to PRRs present on the host cells initiate immune signaling pathways (14). For example, binding of toll-like-receptor (TLR) ligands to the TLRs induces engagement of the adaptor proteins MyD88 or TRIF, followed by further downstream signaling and eventually the activation of transcription factors. PRR ligands such as TLR2 and TLR7 activate signaling pathways that inhibit the Th2-biased immune response and skew the response towards a Th1 profile (15, 16). In the predominantly Th2-skewed environment of the respiratory tract, the use of adjuvants can facilitate this shift, thereby enhancing the generation of high-affinity neutralizing antibodies against RSV (17).

PRR ligands like monophosphoryl lipid A (18), Pam3CSK4 (19), CpG ODN 1826 (20) etc., were shown to skew immune response towards Th1 upon total respiratory tract (TRT) delivery, however, the mucosal responses were not robust. The use of two PRR ligands has been shown to enhance induction of neutralizing antibodies against RSV synergistically (21, 22). For example, the simultaneous use of Pam3CSK4 (TLR2 ligand) and L18-MDP (NOD2 ligand) enhanced neutralizing antibodies against RSV in mice immunized with a virosomal RSV vaccine, and provided protection after RSV challenge without causing enhanced disease (22). Similar results were obtained when L18-MDP (NOD2 ligand) was used together with CpG-ODN-1826 (TLR9 ligand) (21).

The limitation with the use of two adjuvants is that they need not necessarily bind to PRR on the same cell. Recognition of two PRR ligands by a single cell leads to altered molecular programming of innate immune mechanisms resulting in synergistic enhancement of immune response (23). Moreover, approval of vaccines containing two adjuvants might face regulatory barriers. Chimeric adjuvants that are recognized by two PRRs on one cell are available. Examples of these are CL413 and CL429 that are recognized by TLR2/TLR7 and TLR2/NOD2 respectively. The use of these adjuvants has been shown to enhance antigen-specific immune responses after intramuscular or intradermal delivery with an inactivated chikungunya virus vaccine (24).

Induction of mucosal immunity requires antigen administration to mucosal sites. The lung appears to be an efficient target site for mucosal immunization since it has a large surface and harbors effective immune mechanisms (25). Upon immunization or delivery of the vaccine at the respiratory site, airway epithelial cells produce cytokines and chemokines promoting the recruitment of inflammatory cells and directing the adaptive immune response according to stimuli received. Antigen-presenting cells like dendritic cells (DC), present beneath the epithelial layer, and alveolar macrophages, present in the lumen, constantly patrol the large surface area of the lung (26). Upon activation by pathogens, these epithelial cells effectively bypass the steady state anti-inflammatory Th2 responses in the lung and initiate inflammatory Th1 response against the invading pathogen (27).

Accordingly, delivery of VLP to the lungs and use of appropriate adjuvants could be an attractive approach to induce mucosal and systemic responses. Therefore, in this study, we evaluated the effectiveness of the chimeric adjuvants CL413 and CL429 in inducing mucosal and systemic immune response when co-administered with RSV-VLPs by the pulmonary route in mice. A mixture of Pam3CSK4 (TLR2 agonist) with imiquimod (IMQ) (TLR7 agonist) or L18-MDP (NOD2 agonist) adjuvants were used as comparators in the study. Subsequently, mice were challenged with live RSV and protective immune response was evaluated.

## 2 Materials and methods

### 2.1 Preparation of RSV stock and formalin inactivated RSV

HEp-2 cells (ATCC) were grown and maintained in Minimum essential medium (MEM) containing 10% fetal bovine serum (FBS), and 100 I.U/ml penicillin/streptomycin (all reagents from Gibco). The RSV-A2 (VR-1540, ATCC) strain was propagated by infecting HEp-2 cells and stock was prepared as previously described (9). Formalin inactivated RSV (FI-RSV) was prepared by inactivating virus supernatant with formalin at a final dilution of 1:4000 for 3 days at 37 °C as described (28, 29).

### 2.2 Vaccine formulations

RSV-VLPs were produced as described previously using ExpiSf9 cells (Gibco) (29). Briefly, ExpiSf9 cells were co-infected with three recombinant baculoviruses encoding RSV M, G and preFg respectively. Cell suspension was clarified by centrifugation and VLPs were concentrated and purified by sucrose density ultracentrifugation as described. Adjuvanted-VLP formulations were prepared by mixing 5µg of RSV-VLPs with the following combinations: (i) 10µg Pam3CSK4 + 10µg L18-MDP, (ii) 20µg CL413 (iii) 10µg Pam3CSK4 + 10µg Imiquimod, or (iv) 20µg CL429 1-2 hours prior to immunization. Unadjuvanted VLP (5µg) was used as control for the generated immune response. 5µg FI-RSV was adjuvanted with 50µg alum hydroxide prior to immunization was used as control for unfavorable immune response. The formulations were suspended in 40µl PBS for pulmonary immunization and 50µl PBS for intramuscular immunization. All adjuvants were obtained from Invivogen.

### 2.3 Immunization and challenge of mice

*In vivo* experiments in mice were approved by the Institutional Animal Ethics Committee of Bharati Vidyapeeth Medical College, Bharati Vidyapeeth (Deemed to be University), Pune. Female BALB/c mice, 6-7 weeks old were obtained from the Advanced Centre for Treatment, Research and Education in Cancer, India and distributed randomly to have 6 mice per group. All procedures on mice were executed under the influence of isoflurane anesthesia. Immunization was done twice via the intramuscular (i.m.) or pulmonary (p.l.) routes at an interval of three weeks. For i.m. immunizations, the intended dose of unadjuvanted-VLPs or FI-RSV in a total volume of 50µl was injected in the hind legs. For p.l. route, the intended doses of the VLPs without or with adjuvants in 40µl were administered by delivering 20µl in each nostril. Non-immunized or immunized mice were challenged with 10^6^ TCID_50_ RSV by equally distributing 40µl solution over both nostrils 7 days after the second dose. Mice were sacrificed 4 days after challenge under the influence of anesthesia by cervical dislocation.

### 2.4 Sample collection and processing

Blood samples were collected by retro-orbital puncture 21 days after first the dose, 7 days after the second dose and upon sacrifice using capillary tubes. Nose washes and bronchioalveolar lavages (BAL) were collected by making a small incision in the trachea of sacrificed mice, through which 1 ml PBS (pH 7.4) containing complete protease inhibitor was flushed either in the nasal cavity or lungs as described previously (9).

Spleens from mice were collected in complete Iscove’s Modified Dulbecco’s Medium (IMDM, Gibco) supplemented with 100 I.U/ml penicillin/streptomycin, 0.1% beta-mercaptoethanol (2ME, Sigma) and 10% FBS and stored on ice until processed. Spleens were processed individually using 70µm cell strainers (Becton Dickinson). Splenocytes were treated with Ammonium-chloride-potassium (ACK) lysis buffer (Gibco), washed and resuspended in complete IMDM medium and brought to appropriate concentrations of 1x10^7^ cell/ml after counting.

The right lobes of the lungs from each group of mice were pooled together in IMDM supplemented with 100 IU/ml penicillin/streptomycin, 10% FBS without 2ME and stored on ice until processed. Lung tissues were first minced into pieces and incubated in a 30 mm tissue culture dish with 2ml of complete IMDM containing 1mg/ml Collagenase D (Roche) and 10µg/ml DNaseI (Roche) at 37 °C for 1 hr. After incubation, the remaining tissue and all the fluid in the dish were gently disrupted using syringe plunger by passing through a 70µm cell strainer. Lung cells were treated with ACK lysis buffer to remove red blood cells. Dissociated cells were washed and resuspended in complete IMDM medium, counted, and brought to appropriate concentrations of 1x10^7^ cell/ml (30, 31).

A piece of right lobe of the lung was collected in tissue cassettes, fixed in 10% formalin, sectioned and stained using hematoxylin and eosinophil staining (H&E) to study pathology induced after virus challenge. Lung pathology was scored blindly on a 0–4 severity scale by a pathologist for alveolar infiltrates, bronchitis and vasculitis.

### 2.5 Enzyme-linked immunosorbent assay

ELISA was performed on the samples collected from the challenged mice as described previously (9). BAL and nose washes were evaluated for RSV, preFg and G protein-specific IgA and IgG. Serum samples were analyzed for RSV, preFg, G and M protein specific IgG and IgG subtypes (IgG1 and IgG2a) by ELISA. Briefly, 96-well ELISA plates (Thermo Fisher) were coated overnight at 4 °C with 300 ng purified RSV-VLPs or clarified ExpiSf9 cell supernatant containing RSV-preFg, RSV-G or RSV-M proteins. Next, plates were washed using PBS containing 0.05% Tween 20 and blocked for 1 h at 37 °C using PBS containing 5% FBS. Undiluted BAL/nose wash or two-fold serially diluted serum starting at 1:100 dilution was added to the blocked plates, incubated for 1 hr at 37 °C and washed. Conjugated antibodies (anti-mouse-IgG HRP, anti-mouse-IgA-HRP, anti-mouse-IgG2a HRP and anti-mouse-IgG1 HRP; all from SouthernBiotech) were added, incubated for 1 hr at 37 °C and washed. A colorimetric reaction was initiated using Tetramethylbenzidine (TMB) substrate containing hydrogen peroxide (Clinical Science Products Inc.) and the reaction was stopped after 10 min using 2N H2SO4 (Sisco Research Laboratories). Plates were read at 450 nm with filter at 655 nm using a Synergy HTX reader and Gen5 software (BioTek).

### 2.6 Microneutralization assay

Neutralizing antibody titers were determined as described previously (29). Briefly, mice sera were incubated at 56 °C for 30 minutes to inactivate complement factors, two-fold serially diluted in MEM with 2% FBS and incubated with 100 TCID50/ml of RSV diluted in MEM with 2% FBS at 37 °C for 1 hr. After incubation, 20,000 HEp-2 cells in MEM with 2% FBS were added and incubated for 7 days at 37 °C with 5% CO2. After incubation, cells were fixed using 3.7% formaldehyde in PBS and stained with 1% crystal violet. Wells with CPE were counted and neutralizing 50 (NT50) antibody titers were calculated by the Reed-Muench method (32).

### 2.7 IFN-γ and IL-4 ELISpot assay

IFN-γ and IL-4 ELISpot were performed on splenocytes, and cells isolated from lung using a dual color ELISpot kit as per the manufacturer’s instructions (ImmunoSpot, Cellular Technology Ltd.). Briefly, 1x10^6^ pooled lung cells from each group or splenocytes from each mouse were added to PVDF plates coated with a mix of anti-mouse IFN-γ/anti-mouse IL-4 antibodies in complete IMDM medium and stimulated with 1 µg purified VLPs for 18 hr in a humidified 37 °C incubator equilibrated with 5% CO2. Non-stimulated cells were kept as control. Cells were discarded after incubation; plates were washed with PBST, and cytokine specific detection antibodies were added. Blue spots specific for IL-4 and red spots specific for IFN-γ were developed to measure cytokine-specific spot forming cells (SFCs). Spot numbers from non-stimulated wells were subtracted from spots from the stimulated wells to determine antigen-specific cytokine secreting cells after *in vitro* stimulation. Plates were scanned and spots were counted using the ImmunoSpot^®^ Double Color Enzymatic software v7.0.26 (ImmunoSpot).

### 2.8 Cytokine response after immunization

Cytometric bead array (Becton Dickinson) was used to quantify inflammatory (IFN-γ, TNF-α, IL-6, IL-2, IL-17A) and anti-inflammatory cytokines (IL-4, IL-10) secreted by pooled cells from lung or splenocytes as per the manufacturer protocol. Briefly, 1x10^6^ pooled lung cells from each group or 1x10^6^ splenocytes from each mouse were cultured in 96 well U-bottom plates in complete IMDM medium and stimulated with 1µg purified VLPs for 72 hrs. After incubation, the supernatants were collected and used for cytokine determination. Antigen-specific secretion of cytokines was determined by subtracting cytokine levels of non-stimulated cells from those of stimulated cells.

### 2.9 Flow cytometric analysis

1×10^6^ pooled cells from lung and splenocytes obtained on the day of sacrifice were used to determine the T cell and B cell immune responses by flow cytometry from the RSV-VLP immunized mice. A fixable far-red live/dead stain (Invitrogen - excitation maximum of ~633 nm and emission of ~655 nm) was used to gate live cells for analysis. Cellular responses were then analyzed by staining the cells with fluorescence-conjugated antibodies against cellular markers (**Table 1**). Data from stained cells was acquired on the CytoFLEX LX system (Beckman Coulter) and analyzed using CytExpert software (v2.5).

**Table 1:** Antibodies used for T cell and B cell immune response evaluation.

Antibody target	Fluorochrome	Clone	Source
IFN-γ	BV510	XMG1.2	BioLegend
IgM	BV605	RMM-1	BioLegend
IgD	BV605	11-26c.2a	BioLegend
CD19	BV650	6D5	BioLegend
CD4	BV785	RM4-5	BioLegend
CD3	FITC	17A2	BioLegend
Granzyme B	PE/Dazzle 594	QA16A02	BioLegend
CD8a	PerCP	53-6.7	BioLegend
GL7	PE/Cy7	GL7	BioLegend
Ki67	PE	11F6	BioLegend
CD69	APC/Fire 750	H1.2F3	BioLegend
CD103	AF700	2E7	BioLegend
Live/Dead stain	APC	–	Invitrogen

### 2.10 Statistical analysis

Statistical analyses were performed either using the non-parametric two-tailed Mann Whitney test or Kruskal-Wallis test (indicated in figure legends) using Graphpad Prism v10.2.3 (Graphpad Software). Each comparison in the Kruskal-Wallis test was kept stand-alone by applying Dunn’s multiple comparisons test.

## 3 Results

### 3.1 IgA responses in the nasal cavity

**Figure 1:**
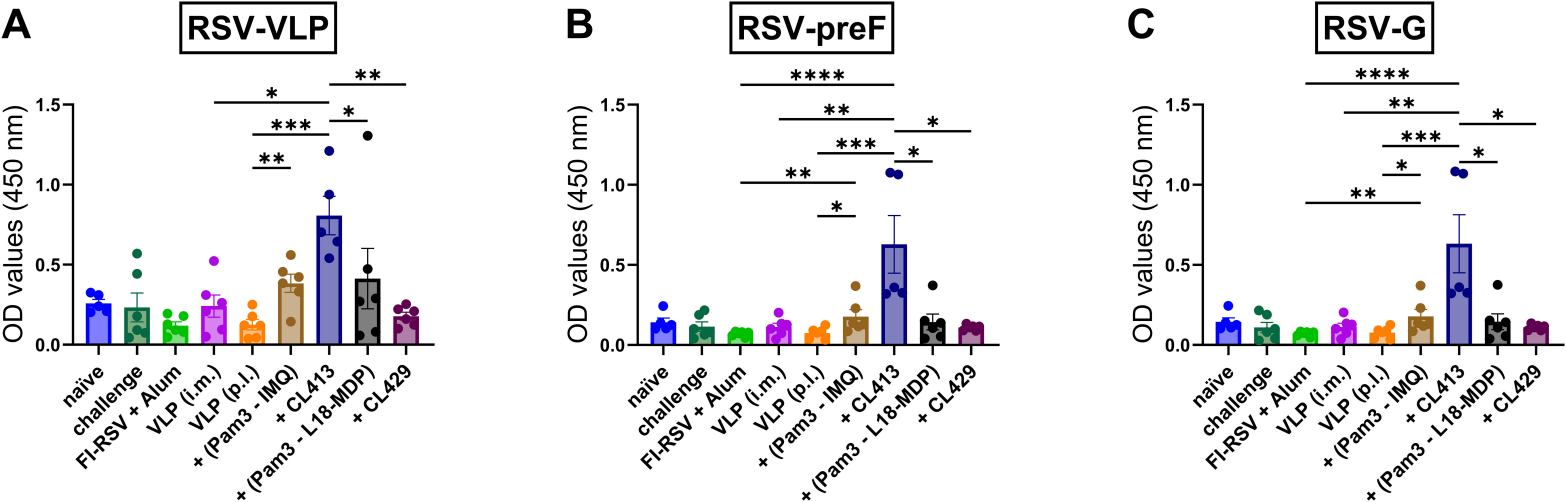
IgA antibody responses in the nasal cavity. Mice (n = 6) were left untreated (naïve) or were immunized twice on day 1 and day 21 with 5 μg of alum adjuvanted FI-RSV (i.m. route), 5 μg unadjuvanted VLPs (i.m. route), 5 μg unadjuvanted VLPs (p.l. route) or 5 μg VLPs adjuvanted with either of Pam3CSK4+L18-MDP, Pam3CSK4+IMQ, CL413 or CL429 (administered via the p.l. route). Immunized mice were challenged with wild-type-live RSV seven days after the second dose (day 28) and sacrificed four days after challenge (day 32). Upon sacrifice, the nasal cavity was flushed with 1 ml PBS containing protease inhibitor. Undiluted nose wash was added directly to ELISA plates to determine the induction of IgA antibodies against **(A)** RSV-VLPs, **(B)** RSV-preF, and **(C)** RSV-G proteins. Statistical analysis was accomplished using the non-parametric Kruskal-Wallis test: *p < 0.05; **p < 0.01; ***p < 0.001; ****p < 0.0001. Bar represents mean ± SEM.

IgA in the nasal cavity has been shown to be associated with protection at the mucosal surfaces (33–36). Therefore, the induction of IgA antibodies in the nasal cavity was accessed to evaluate whether adjuvanted RSV-VLPs elicited IgA in the nasal cavity after pulmonary immunization (**Figures 1A–C**). Only mice immunized with CL413 adjuvanted VLPs induced significantly higher IgA levels than the unadjuvanted VLPs (**Figure 1A**), preF (**Figure 1B**) and G proteins (**Figure 1C**). No IgA induction was observed when VLPs were administered alone or adjuvanted with combinations of separate adjuvants (Pam3CSK4+IMQ or Pam3CSK4+L18-MDP) or CL429. As expected, IgG-anti-RSV was not detected in the nasal cavity after pulmonary immunization (data not shown).

### 3.2 IgA and IgG responses in the lungs

**Figure 2:**
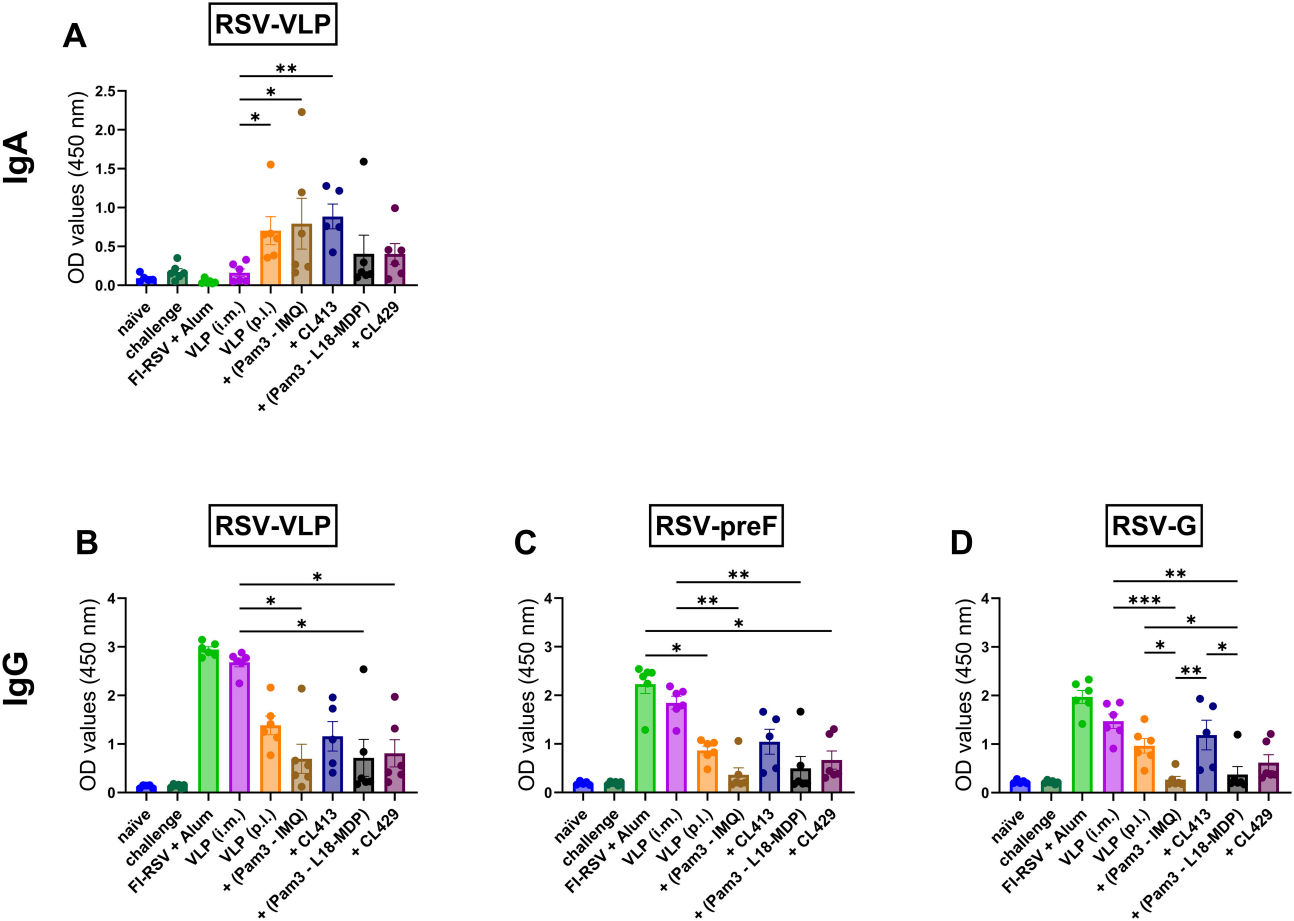
Antibody responses in the lungs. Mice (n = 6) were immunized with two doses with non-adjuvanted or adjuvanted RSV-VLPs as described previously and sacrificed 4 days after challenge with wild-type live RSV. Upon sacrifice, the lungs were flushed with 1 ml PBS containing protease inhibitor. Undiluted lung washes were added directly to ELISA plates to determine the induction of **(A)** IgA antibodies against RSV-VLPs and IgG antibodies against **(B) **RSV-VLP, **(C)** preF and **(D)** RSV-G proteins. Statistical analysis was accomplished using the non-parametric Kruskal-Wallis test: *= p < 0.05; **= p < 0.01; ***= p < 0.001. Bar represents mean ± SEM.

RSV primarily affects the lung, therefore protection of the lung from RSV is essential. Both IgA and IgG are shown to neutralize the viruses in the lungs (37, 38). Next, we evaluated the levels of these antibodies post- immunization in the lung washes. Intramuscular immunization with VLPs alone failed to elicit BAL-IgA. In contrast, when administered via the pulmonary route, unadjuvanted VLPs, VLPs adjuvanted with Pam3CSK4+IMQ or CL413 induced substantial IgA levels. Mice immunized with Pam3CSK4+L18-MDP or CL429 adjuvanted VLPs minimally induced IgA in the lungs (**Figure 2A**).

Evaluation of IgG in BAL showed that i.m. administered unadjuvanted VLPs and alum-adjuvanted FI-RSV administered via the i.m. route induced similar levels of IgG against the VLPs (p = 0.55) (**Figure 2B**), preF (p = 0.62) (**Figure 2C**) and G proteins (p = 0.42) (**Figure 2D**) The IgG levels against the i.m. administered VLPs were higher than those induced by either of the adjuvanted formulations delivered by the pulmonary route (**Figures 2B–D**). No difference in IgG levels against VLPs in the BAL was observed after pulmonary delivery of unadjuvanted and adjuvanted VLPs.

Among the pulmonary immunized mice, the VLP+CL413 combination consistently induced higher IgG levels against preF and G proteins than the other adjuvanted formulations. Surprisingly, reduction in IgG antibody induction was observed as compared to unadjuvanted VLPs in the BAL of mice immunized with other adjuvanted formulations. The CL413 adjuvant induced higher IgG-anti-preF (p = 0.051) (**Figure 2C**) and -anti-G levels (p = 0.06) (**Figure 2D**) than Pam3CSK4+IMQ combination adjuvants. Contrarily, the levels of IgG induced by CL429 and Pam3CSK4+L18-MDP combination adjuvants towards preF (p = 0.33) and G (p = 0.15) proteins were similar.

### 3.3 Systemic humoral immune responses

**Figure 3:**
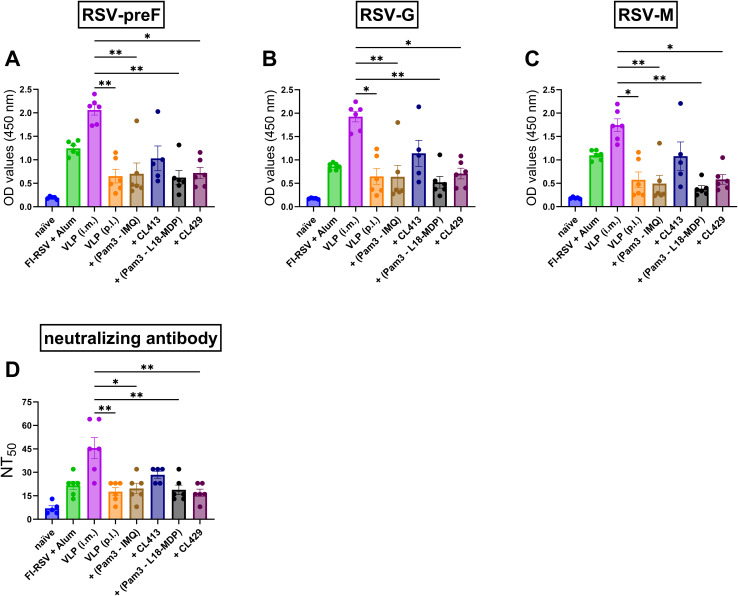
Systemic humoral immune response after immunization. Mice (n = 6) were immunized with two doses of the non-adjuvanted or adjuvanted RSV-VLPs as described previously. Sera were collected seven days post second dose (day 28). Humoral responses were determined by measuring IgG antibodies against **(A)** RSV-preF **(B)**RSV-G, **(C)** RSV-M proteins and **(D)**RSV-neutralizing antibody titers in the sera. Statistical analysis was accomplished using the non-parametric Kruskal-Wallis test: *= p < 0.05; **= p < 0.01. Bar represents mean ± SEM.

To understand the systemic antibody generated after immunization with adjuvanted-VLPs via the pulmonary route, binding IgG antibody levels (**Figures 3A–C**) against preF, G and M; and RSV-neutralizing antibody levels (**Figure 3D**) were measured in the serum samples obtained post second dose. Except for CL413-adjuvanted VLPs, all the other formulations induced similar levels of IgG-anti-preF (p = 0.73 to 0.99) (**Figure 3A**), -anti-G (p = 0.64 to 0.80) (**Figure 3B**) and -anti-M (p = 0.68 to 0.73) (**Figure 3C**) proteins as those induced after pulmonary delivery of the unadjuvanted VLPs. CL413 adjuvanted VLPs, administered p.l., and alum-adjuvanted FI-RSV, administered i.m., induced comparable IgG levels against all the three proteins (p = 0.44 to 0.77) (**Figures 3A–C**). These levels were lower than those induced by i.m. administered VLPs, but this difference was not statistically significant. In contrast, all other p.l. delivered formulations were inferior to those induced after intramuscular delivery of the VLPs. Consistent with RSV-anti-IgG levels, CL413 adjuvanted VLPs, alum-adjuvanted FI-RSV and i.m. delivered unadjuvanted VLPs induced comparable virus neutralizing antibody levels (p = 0.24 to 0.49) (**Figure 3D**).

### 3.4 Systemic IgG2a and IgG1 subtype responses

**Figure 4:**
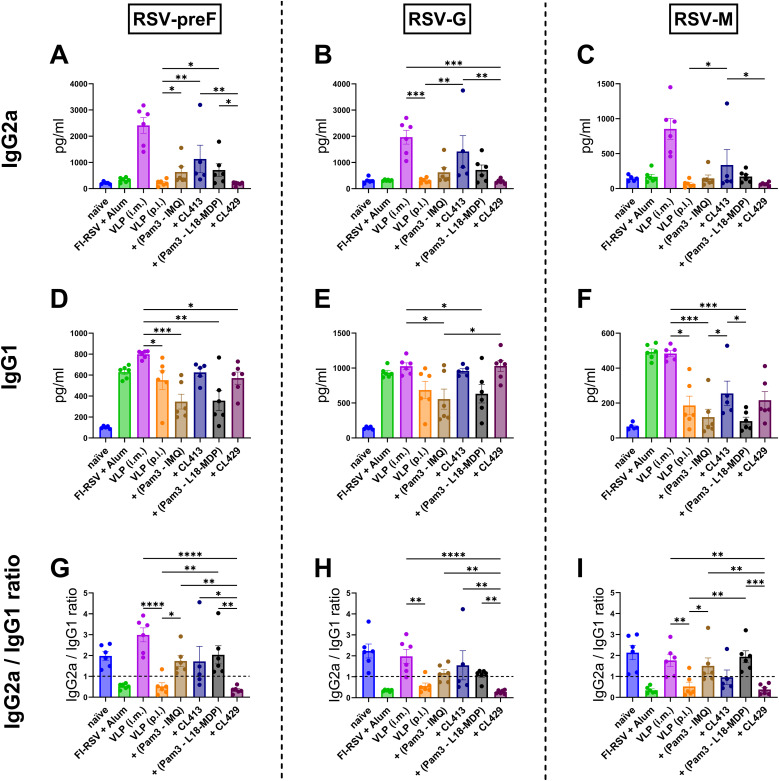
Antibody subtype responses after immunization with adjuvanted RSV-VLPs. Mice (n = 6) were immunized with two doses of the adjuvanted or adjuvanted RSV-VLPs as described previously. Sera obtained from mice seven days post the second dose (day 28) were analyzed for IgG subtypes using RSV-preF, RSV-G and RSV-M proteins for coating the ELISA plates (IgG2a **(A–C)** and IgG1 **(D–F)**). The ratios of IgG2a to IgG1 for antibodies to **(G)** preF, **(H)** G, and **(I)** M proteins were calculated for individual mice. Horizontal dotted line indicates IgG2a/IgG1 ratio = 1. Statistical analysis was accomplished using the non-parametric Kruskal-Wallis test: *= p < 0.05; **= p < 0.01; ***= p < 0.001; ****= p < 0.0001. Bar represents mean ± SEM.

Understanding the IgG subtype response, IgG1 and IgG2a, which serve as markers of Th1 or Th2 immune bias response is crucial for RSV vaccines, as it influences efficacy and the potential risk of vaccine-enhanced disease (17). Therefore, antibody subtype responses against F, G and M proteins were characterized by measuring IgG2a (**Figures 4A–C**) and IgG1 (**Figures 4D–F**) subtype antibodies (39).

As expected, mice immunized with alum-adjuvanted FI-RSV failed to elicit IgG2a (**Figures 4A–C**), while mice receiving VLPs intramuscularly produced the highest levels of IgG2a. When pulmonary delivery was considered, CL413-adjuvanted VLPs elicited significantly enhanced IgG2a production against all the constituent proteins compared to the unadjuvanted VLPs (p = 0.04 to 0.009). IgG2a levels in the mice of the CL413 group were lower than those in the i.m. VLP group, though the difference was not statistically significant (p = 0.42 to 0.58). IgG2a induction was also observed in mice immunized with a combination of Pam3CSK4 with L18-MDP or imiquimod. CL429-adjuvanted VLPs failed to induce IgG2a upon pulmonary immunization.

Evaluation of IgG1 (**Figures 4D–F**) highlighted that, unlike IgG2a, alum adjuvanted FI-RSV induced IgG1. The levels of IgG1 elicited after immunization with VLPs via the intramuscular or pulmonary route were comparable to those induced by alum-adjuvanted FI-RSV. An adjuvant effect of both CL413 and CL429 was observed for IgG1 against preF and G proteins; however, the IgG1 increase was marginal as compared to the unadjuvanted VLPs. The intramuscular route was superior to pulmonary delivery when IgG1 levels against the M protein were considered.

IgG2a/IgG1 ratio (**Figures 4G–I**) demonstrated that as previously reported, alum-adjuvanted FI-RSV induced skewed IgG1 response against all proteins. Similarly, pulmonary immunization with unadjuvanted VLPs induced predominantly IgG1 antibodies. Contrarily, except for CL429, VLPs adjuvanted with adjuvants resulted in an IgG2a skewed antibody response against all the proteins upon p.l. delivery.

### 3.5 B cell responses after immunization

Ideally, RSV vaccines should generate robust mucosal and systemic B cells to overcome the defective responses seen after infection with live virus (40–43). To address this important issue, we evaluated the B cells in the lungs and spleens upon immunization. The gating strategy for the flowcytometric analysis of B cell responses is summarized in **Supplementary Figure S1**.

**Figure 5:**
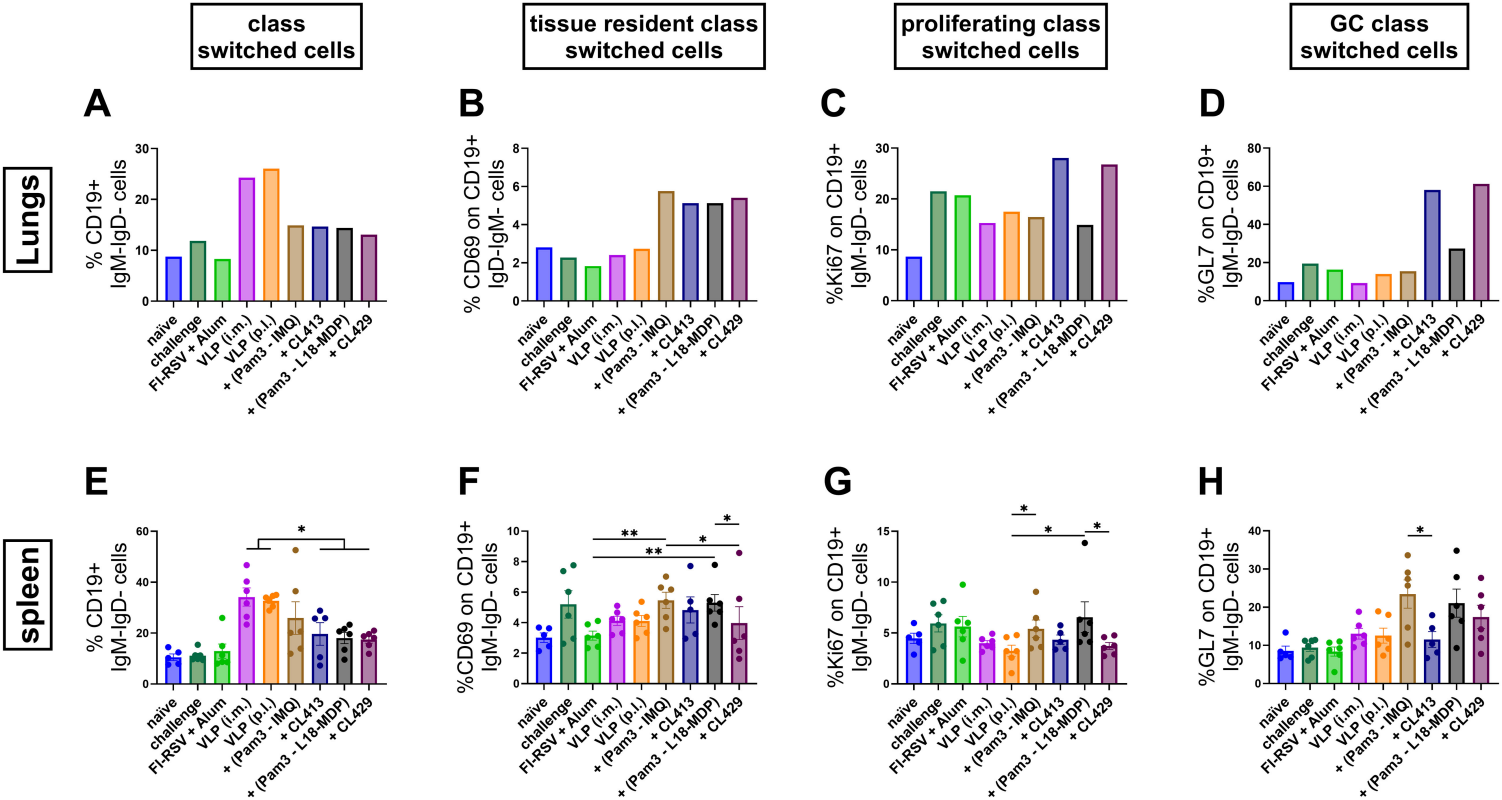
Class-switched B cell response after immunization. The class-switched B cell response was evaluated by multicolor flowcytometry using lung lymphocytes and splenocytes of immunized mice (n = 6) that were sacrificed four days after challenge following the second immunization dose (day 32). **(A, E)** Levels of class switched B cells, **(B, F)** tissue resident class switched B cells, **(C, G)** germinal center class switched B cells and **(D, H)** proliferating class switched B cells after immunization were determined. Lung lymphocytes from each group were pooled and used for the assay. Splenocytes from each mouse were processed and analyzed individually. Statistical analysis was accomplished using the non-parametric Kruskal-Wallis test: *p < 0.05; **p < 0.01; ***p < 0.001. Bar represents mean ± SEM.

Post-intramuscular immunization with FI-RSV or the unadjuvanted VLPs, higher percentages of class-switched B cells (CD19+IgM-IgD-) were observed in the lungs, consistent with the elevated IgG antibody levels in the lungs or serum, as compared to the p.l. administered unadjuvanted or adjuvanted VLPs (**Figure 5A**). On the contrary, all the adjuvants elicited similar levels of resident class switched B cells in the lungs (CD19+IgM-IgD-CD69+) (**Figure 5B**). The chimeric adjuvants enhanced the proliferation capacity of the class-switched cells (CD19+IgM-IgD-Ki67+) by almost two-fold compared to non-adjuvanted i.m. or p.l. delivered VLPs (**Figure 5C**).

While the majority of class-switched B cells were found in both the lungs and spleens after i.m. immunization, the chimeric adjuvants, CL413 and CL429, notably resulted in the highest numbers of proliferating (CD19+IgM-IgD-Ki67+, **Figure 5C**) and GC-resident (CD19+IgM-IgD-GL7+, **Figure 5D**) B cells in the lungs, further highlighting the ability of these adjuvants to promote enhanced B cell responses. The presence of class-switched GC B cells in the lungs was increased by almost four-fold with CL413 and CL429 compared to the unadjuvanted VLPs delivered via either the i.m. or p.l. routes or VLPs combined with Pam3CSK4 and L18-MDP or IMQ (**Figure 5D**). The combination adjuvants increased GC reactions by two-fold compared to the unadjuvanted VLPs (p = 0.16 to 0.25). CL413 adjuvantation had no effect on the class switched GC B cells in the spleens (**Figure 5H**).

Surprisingly, we found a trend towards an increased percentage of resident marker on the class switched B cells in the spleens after delivery of adjuvanted VLP to the lungs (**Figure 5F**). Even, except for CL413, increased number of GL7+ cells were induced in spleens by adjuvanted VLPs (**Figure 5G**).

### 3.6 IFN-γ and IL-4 response in the lungs and spleens

**Figure 6:**
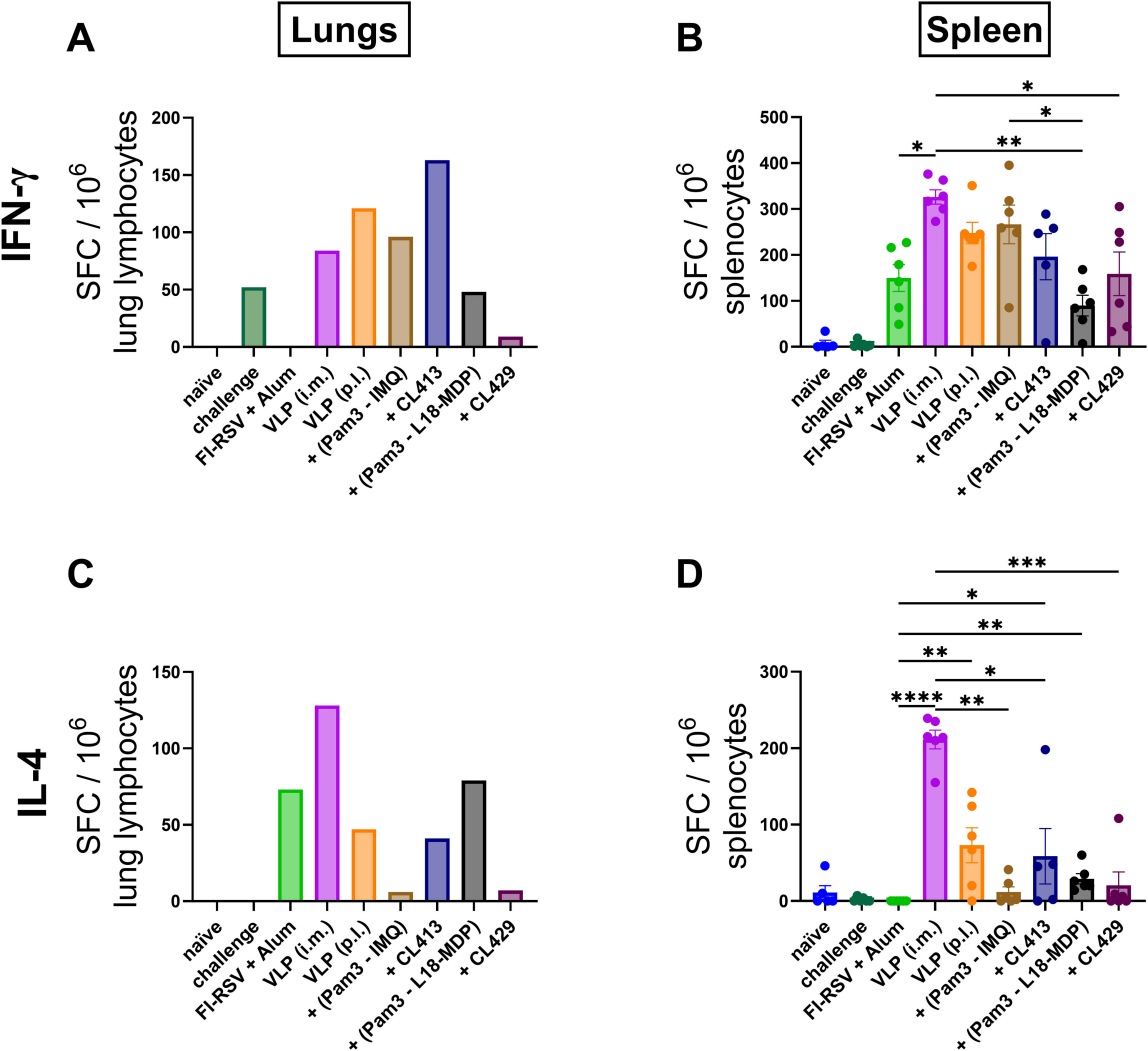
In vitro cytokine production. IFN-γ and IL-4 spot forming cells (SFCs) were quantified from lymphocytes from lungs and spleens of immunized mice (n = 6) that were sacrificed 4 days after challenge (day 32). Lung lymphocytes from each group were pooled and used for the assay. Splenocytes from each mouse were cultured separately. The cells were cultured either in presence or absence of purified VLPs for 18hrs. Antigen-specific **(A, B)** IFN-γ and **(C, D)** IL-4-SFC were determined by subtracting spot numbers of the non-stimulated wells from those of the stimulated wells. Statistical analysis was accomplished using the non-parametric Kruskal-Wallis test: *p < 0.05; **p < 0.01; ***p < 0.001; ****p < 0.0001. Bar represents mean ± SEM.

We next determined the antigen specific IFN-γ (**Figures 6A, B**) and IL-4-SFC (**Figures 6C, D**) post immunization. All the immunized mice, except those immunized with FI-RSV, harbored RSV-specific IFN-γ-SFC in the lungs (**Figure 6A**). When pulmonary delivery was considered, a distinct difference was observed in the number of IFN-γ-SFCs in the lungs of the immunized mice; lowest in the group immunized with VLPs+CL429 and highest with VLPs+CL413. Notably the induction of IFN-γ-SFC was also observed in the lungs of the naïve mice that were challenged with wild-type-RSV.

Evaluation of the splenocytes highlighted that all immunized mice produced RSV-specific IFN-γ-SFCs (**Figure 6B**), the numbers being comparable among the mice immunized p.l. with unadjuvanted VLPs, VLP+Pam3CSK4+IMQ and VLP+CL413 and the mice immunized with unadjuvanted-VLPs via the intramuscular route (p = 0.17 to 0.43). In contrast, the mice immunized with FI-RSV, VLP+Pam3-L18-MDP and VLP+CL429 produced a significantly lower number of IFN-γ-SFCs than mice intramuscularly immunized with the unadjuvanted VLPs (p = 0.001 to 0.02). Pulmonary delivery of VLPs combined with CL413 induced similar numbers of IFN-γ-SFC in both lungs and spleen, while the other immunized mice induced lower IFN-γ-SFCs in the lungs and higher in the spleen.

Evaluation of antigen-specific IL-4 in lungs (**Figure 6C**) and spleens (**Figure 6D**) highlighted that the pulmonary administered, unadjuvanted or adjuvanted VLPs induced lower numbers of IL-4-SFC than VLPs delivered by the intramuscular route. The pulmonary route led to lower IL-4-SFC numbers for VLPs adjuvanted with Pam3CSK4+IMQ or CL429 than for VLPs alone. Additionally, mice administered with Pam3CSK4+L18-MDP or CL413 produced IL-4-SFC numbers comparable to those produced by unadjuvanted VLP delivered by the pulmonary route. Surprisingly, mice immunized with alum-adjuvanted FI-RSV did not induce antigen-specific IL-4-SFC in the splenocytes in response to the *in vitro* stimulation, however, these mice had the overall highest number of stimulated and non-stimulated IL-4-SFCs (456 ± 20 SEM and 400 ± 22 SEM).

### 3.7 Inflammatory and anti-inflammatory cytokine responses in lungs and spleens

**Figure 7:**
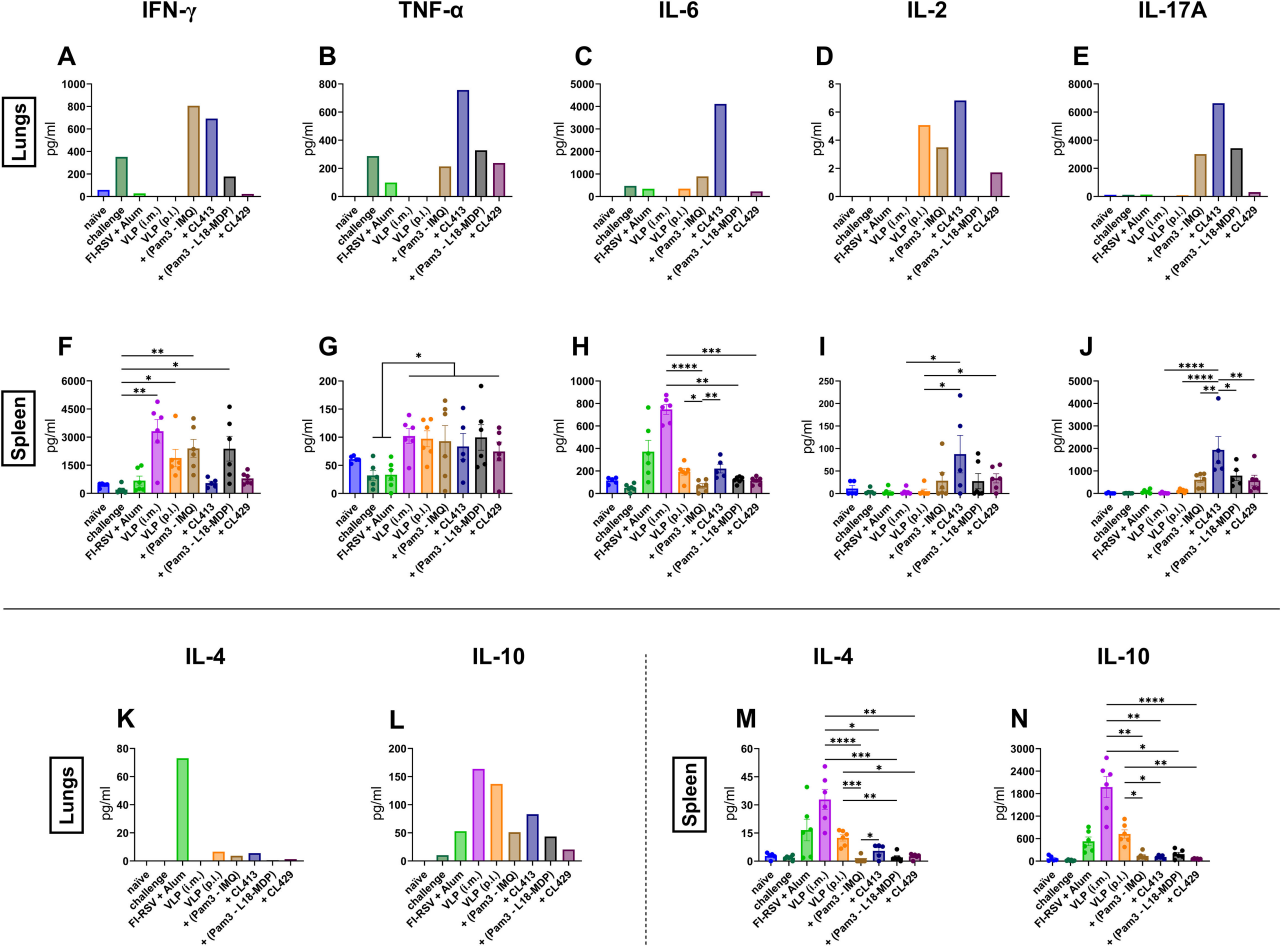
Inflammatory and anti-inflammatory cytokine responses after immunization. Mice (n = 6) were sacrificed 4 days after challenge following the second immunization dose. Lung lymphocytes from one group were pooled and used for the assay. Splenocytes from each mouse were cultured separately. The cells were cultured either in the presence or absence of purified VLPs for 18hrs. Supernatants were used to determine **(A, F)** IFN-γ, **(B, G)** TNF-α, **(C, H)** IL-6, **(D, I)** IL-2, **(E, J)** IL-17A, **(K, M)** IL-4 and **(L, N)** IL-10 from non-stimulated and stimulated cells. Antigen-specific responses were determined by subtracting values of the cytokines of non-stimulated cells from those of the stimulated cells. Statistical analysis was accomplished using the non-parametric Kruskal-Wallis test: *p < 0.05; **p < 0.01; ***p < 0.001; ****p < 0.0001. Bar represents mean ± SEM.

Cytokines play a key role in shaping the immune response by secretion of various cytokines in response to RSV infection (44). We next evaluated the antigen-specific inflammatory (**Figures 7A–J**) and anti-inflammatory (**Figures 7K–N**) cytokine responses after *in vitro* stimulation of the lung lymphocytes and splenocytes of the immunized mice.

Intramuscular administration of the unadjuvanted VLPs or the alum-adjuvanted FI-RSV failed to elicit antigen-specific inflammatory cytokines responses in lung lymphocytes (**Figures 7A–E**). VLP+CL413 induced the highest levels of pro-inflammatory cytokines in the lung cells as compared to the other adjuvants (**Figures 7A–E**). Pam3CSK4+IMQ-adjuvanted VLPs also induced elevated levels pro-inflammatory cytokines but only IFN-γ levels were equivalent to that induced by VLP+CL413 in the lung lymphocytes. VLPs combined with Pam3CSK4+L18-MDP were able to induce only TNF-α and IL17-A after *in vitro* stimulation. Immunization with VLP+CL429 elicited minimal levels of all pro-inflammatory cytokines in the lung cells.

Analysis of anti-inflammatory cytokines from the lung cells highlighted that the mice immunized with alum-adjuvanted FI-RSV produced about thirty-fold more IL-4 (**Figure 7K**) as compared to pulmonary immunized mice. Although no difference was seen in IL-10 levels (**Figure 7L**) between FI-RSV immunized mice and mice immunized by the adjuvanted VLPs, delivery of unadjuvanted VLP by intramuscular or pulmonary route produced two-to-three-fold more IL-10 than found in the other groups. Overall, the VLPs combined with the CL413 adjuvant induced proinflammatory cytokines in the lungs that are associated with favorable immune response after immunization.

Next, we compared the secretion of cytokines by the stimulated splenocytes in the culture supernatants. TNF-α levels were independent of the delivery route of the VLPs (**Figure 7G**). The levels of the pro-inflammatory cytokines IFN-γ (**Figure 7F**) and IL-6 (**Figure 7H**) and the anti-inflammatory cytokines IL-4 (**Figure 7M**) and IL-10 (**Figure 7N**) were higher in splenocytes of intramuscularly immunized mice than in splenocytes of the other groups. The use of the adjuvant CL413 for pulmonary delivery induced the highest levels of IL-2 (**Figure 7I**) and IL-17A (**Figure 7J**) in the splenocytes after *in vitro* stimulation as compared to the use of the other adjuvants. Overall, these results highlight that CL413 plays a crucial role in inducing cytokine response in the induction of cytokine-producing lymphocytes in the lung. Moreover, it promotes IL-2 and IL-17A production by splenocytes, cytokines that are known to play a critical role in mucosal response induction (45–47).

### 3.8 CD4+ T cell responses after immunization

**Figure 8:**
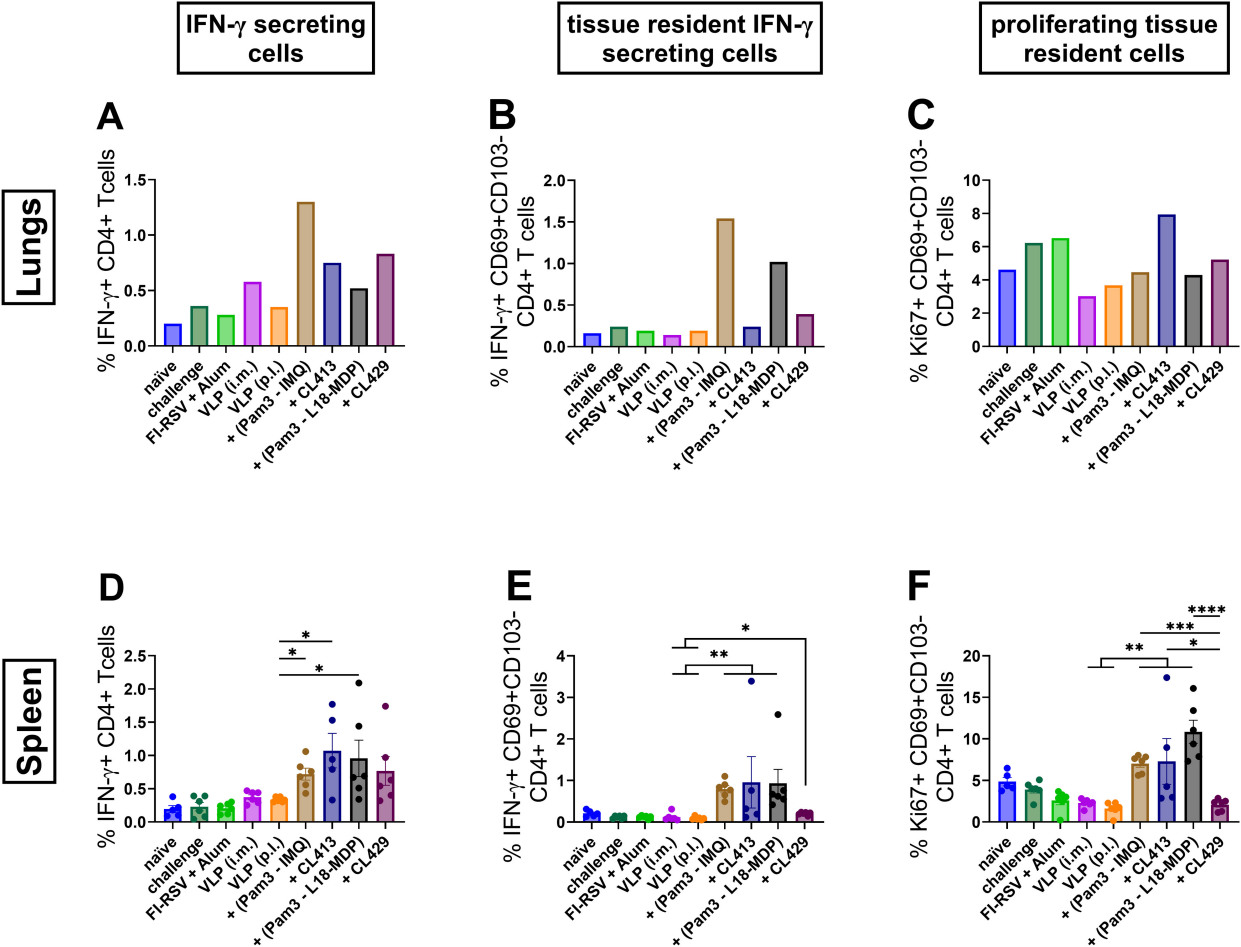
CD4+ T cell responses after immunization. Lymphocytes from lungs or spleens of immunized mice (n = 6) that were sacrificed 4 days after challenge following the second immunization dose were subjected to flow-cytometry analysis to determine **(A, D)** IFN-γ positivity among CD4+ T cells, **(B, E)** IFN-γ positivity among tissue resident T cells and **(C, F)** proliferating among tissue resident CD4+ T cells. Lung lymphocytes from each group were pooled and used for the assay. Splenocytes from each mouse were analyzed individually. Statistical analysis was accomplished using the non-parametric Kruskal-Wallis test: *p < 0.05; **p < 0.01; ***p < 0.001; ****p < 0.0001. Bar represents mean ± SEM.

The T cell response to RSV infection plays a critical role in mediating both viral clearance and a decrease in disease severity (48, 49). Therefore, we evaluated the CD4+ T cell response in the lungs and spleens of the immunized and challenged mice. The gating strategy for the flowcytometric analysis of CD4+ T cell responses is summarized in **Supplementary Figure S1**. All the mice immunized with adjuvanted formulations, except Pam3CSK4+L18-MDP induced a higher percentage of IFN-γ-secreting CD4+ T cells in the lungs as compared to the unadjuvanted VLPs delivered either by i.m. or the p.l. route (**Figure 8A**). Similarly, all the mice immunized with adjuvanted formulations had a significantly higher percentage of these cells in the spleen (**Figure 8D**).

Tissue resident cells play a critical role in the local defense against respiratory infections (50). Lung tissue resident cells are defined as T cells expressing phenotypic markers including CD69 for both CD4+ and CD8+ T cells, and CD103 for CD8+ T cells (50). Further analysis of these cells for their capacity to reside in the lungs highlighted that the chimeric adjuvants, CL413 and CL429, minimally promote tissue resident CD4+ T cells that can secret IFN-γ (CD4+CD69+CD103-IFN-γ+) in the lungs (**Figure 8B**), but significantly higher compared to the unadjuvanted VLPs (p 0.01 to 0.05). This capacity, however, was significantly lower in comparison to Pam3+L18-MDP and Pam3-IMQ adjuvanted formulations in the spleens (**Figure 8E**).

Further evaluation of CD4+ resident T cells highlighted that only CL413 promoted development of CD4 tissue resident cells with proliferative capacity (CD4+CD69+CD103-Ki67+) in the lungs (**Figure 8C**). On the contrary, all adjuvants, except CL429, induced significantly higher resident cells having the capacity to proliferate in the spleen (**Figure 8F**) as compared to the unadjuvanted VLPs.

### 3.9 CD8+ T cell responses after immunization

CD8+ T cells are important in eliminating virus-infected cells in the respiratory tract (51, 52). Therefore, we evaluated the CD8+ T cell response after immunization in the lungs and spleen. The gating strategy for the flowcytometric analysis of CD8+ T cell responses is summarized in **Supplementary Figure S1**.

**Figure 9:**
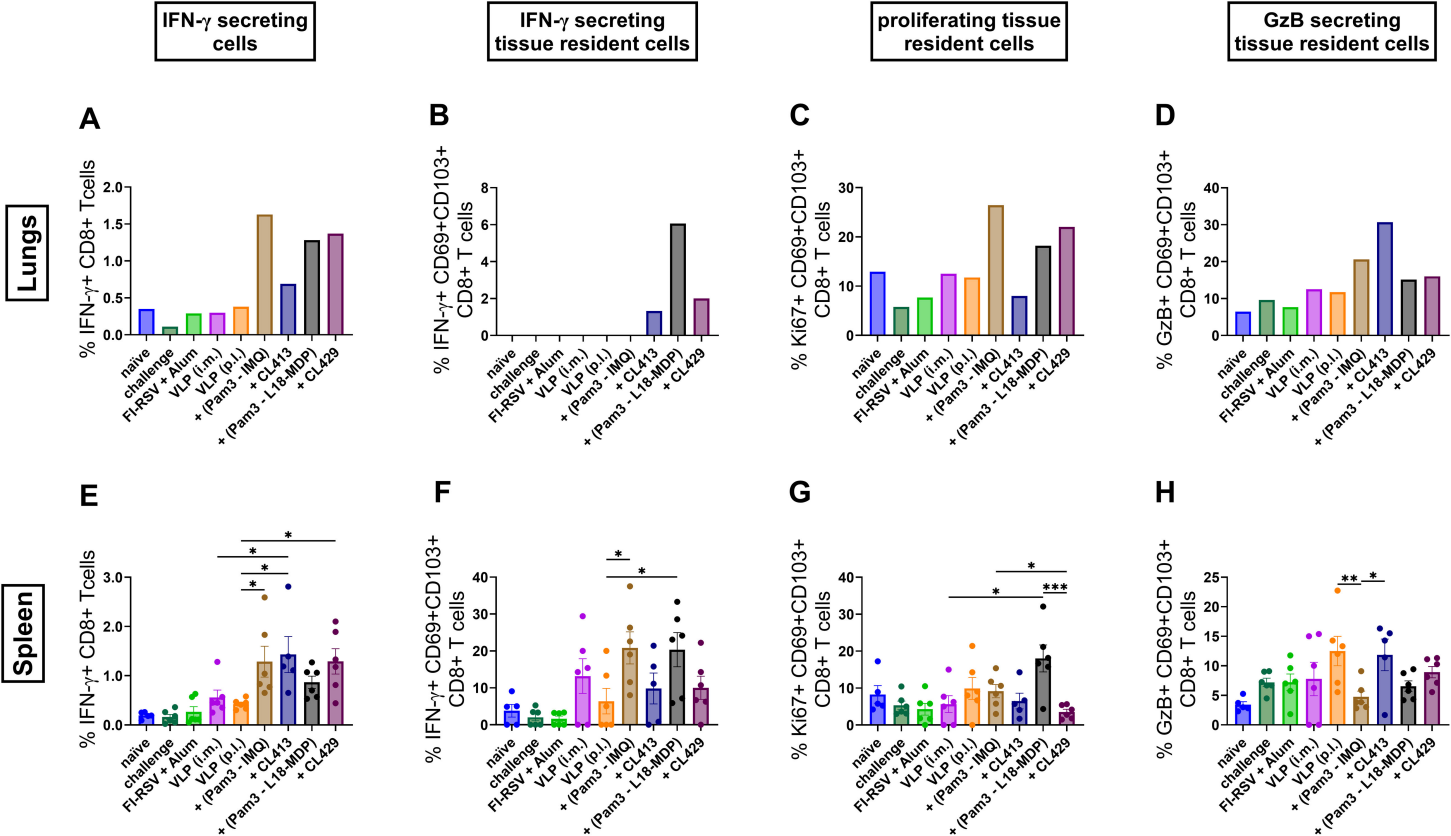
CD8+ T cell responses after immunization. Lymphocytes from lungs or spleens of immunized mice (n = 6) that were sacrificed 4 days after challenge following the second immunization dose were subjected to flow-cytometry analysis to determine **(A, E)** CD8+ T cells producing IFN-γ, **(B, F)** tissue resident CD8+ T cells having the capacity to secrete IFN-γ, **(C, G)** proliferating tissue resident CD8+ T cells and **(D, H)** granzyme B positivity among tissue resident CD8+ T cells. Lung lymphocytes from each group were pooled and used for the assay. Splenocytes from each mouse were analyzed individually. Statistical analysis was accomplished using the non-parametric Kruskal-Wallis test: *p < 0.05; **p < 0.01; ***p < 0.001; ****p < 0.0001. Bar represents mean ± SEM.

Mice immunized with VLPs combined with Pam3CSK4+L18-MDP, Pam3CSK4+IMQ, CL413 and CL429 showed a higher number of IFN-γ secreting CD8+ T cells (CD8+IFN-γ+) in both lungs and spleens (p = 0.03 to 0.04) as compared to the other immunized mouse groups (**Figures 9A, E**). Among tissue resident CD8+ T cells (CD8+CD69+CD103+IFN-γ+), IFN-γ positivity was observed in mice immunized with Pam3CSK4+L18-MDP-, CL413- and CL429-adjuvanted VLPs in the lungs (**Figure 9B**), while the highest percentages in the spleens were induced by Pam3CSK4+L18-MDP- and Pam3CSK4-IMQ-adjuvanted VLPs (**Figure 9B**).

All adjuvanted formulations except CL413, induced proliferating tissue resident CD8+ T cells in the lungs characterized by expression of Ki67 (**Figure 9C**). This adjuvant effect was limited in the spleens (**Figure 9G**). Pam3CSK4+IMQ adjuvanted VLPs induced the highest levels of proliferating tissue resident CD8+ T (CD8+CD69+CD103+Ki67+), cells in the lungs (**Figure 9C**) and Pam3CSK4+L18-MDP induced the highest levels of proliferating tissue resident CD8+ T cells in the splenocytes (**Figure 9G**).

Granzyme B is a protease that is released by cytotoxic T lymphocytes (CTLs) and NK cells and contributes to cell death of virus infected cells (53, 54). When we evaluated granzyme secretion in the tissue resident CD8+ T cells (CD8+CD69+CD103+GzB+), we found that CL413 increases granzyme B secretion by three-to-four-folds in the lungs as compared to the i.m. or p.l. delivered unadjuvanted VLPs (**Figure 9D**). Likewise, adjuvant effects were also seen with the other adjuvant groups wherein granzyme B secretion was moderately increased in these cells. This beneficial effect of using adjuvants was not observed when the granzyme B secretion was evaluated in the tissue resident CD8+ T cells from the splenocytes (**Figure 9H**).

### 3.10 Protection from RSV after challenge

**Figure 10:**
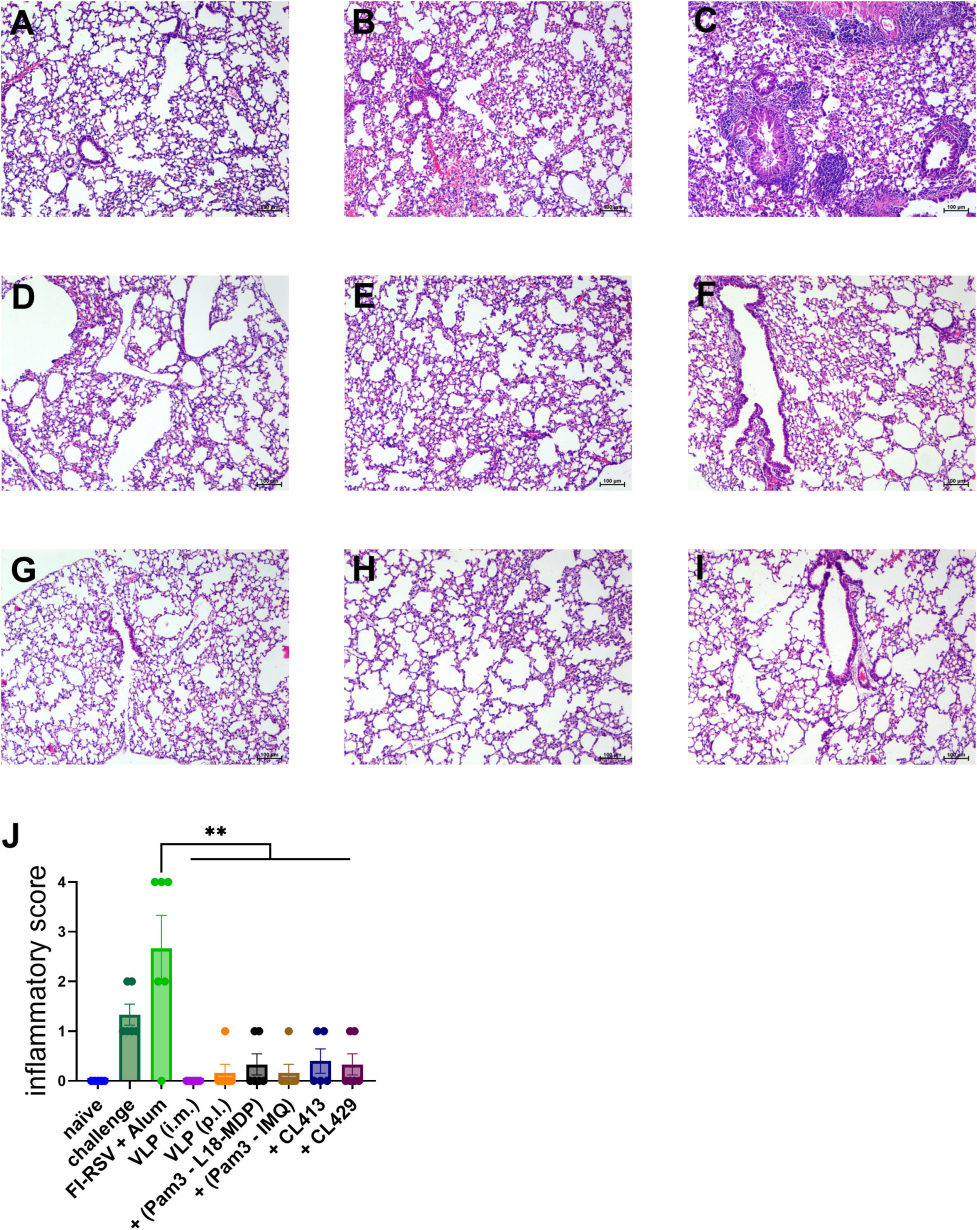
Protection from lung pathology after immunization and RSV challenge. Immunized mice (n = 6) were challenged with wild type-live RSV and lungs were harvested 4 days after challenge. Lungs from all the mice were fixed, sectioned, and stained with H&E to evaluate RSV-mediated pathology. Representative images of the H&E-stained lung sections from **(A)** naïve, **(B)** non-immunized and challenged **(C)** alum-adjuvanted FI-RSV, **(D)** intramuscularly administered VLPs, pulmonary administered **(E)** VLPs, **(F)** VLP+Pam3CSK4+L18-MDP **(G)** VLP+Pam3CSK4+IMQ, **(H)** VLP+CL413 and **(G)** VLP+CL429 mice. The lung pathology score was calculated after analyzing the lung sections from each mouse. Statistical analysis was accomplished using the non-parametric Kruskal-Wallis test: **= p < 0.01. Bar represents mean ± SEM.

To investigate whether mucosal delivery of RSV-VLPs combined with adjuvants protects the mice from RSV induced lung pathology, we examined the lungs from the mice four days after challenge RSV-challenged seven days post the second immunization (**Figures 10A–I**). No lung pathology was observed in the challenged mice immunized by intramuscular route. Importantly, the signs of alveolitis and infiltrates in bronchial and vascular areas were not observed in the mice immunized with unadjuvanted or adjuvanted VLPs. The lung pathology score was comparable to the naive mice (**Figure 10A**). Similar to the previous results, only mice immunized with FI-RSV showed signs of enhanced inflammation after challenge (**Figure 10B**).

## 4 Discussion

**Figure 11:**
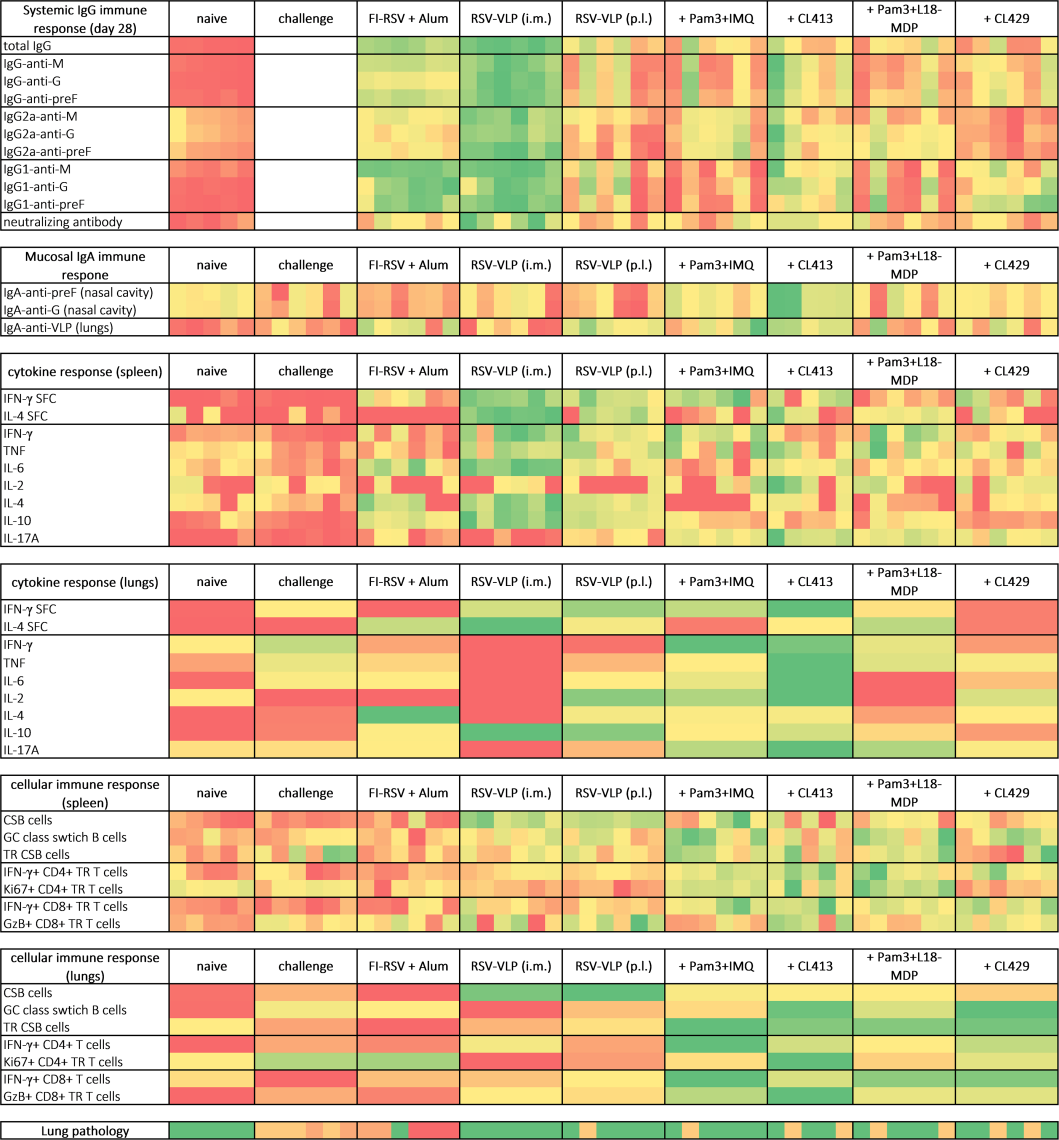
Heat map of the key immune responses induced after immunization and challenge with RSV. Mice were immunized, challenged and immune responses were evaluated as indicated in the materials and methods section. Heat map was plotted by using the generated data as input in MS Excel and applying conditional formatting. Each column represents one animal of systemic IgG response, mucosal IgA response, cytokine/cellular response in spleens and lung pathology. Lung lymphocytes for each group were pooled together to determine cytokine and cellular response in lungs. For all the parameters, heatmaps range from red (lowest response) to green (best response). CSB and TR depicts class switched B cells and tissue resident cells respectively.

In continuation with our previous study (9), we evaluated pulmonary administration of the adjuvanted preFg-based RSV-VLPs in inducing mucosal and systemic immunity, and protection from RSV-induced inflammation upon RSV challenge (**Figure 11**). Dual pattern recognition receptor agonists CL413 and CL429 were selected as adjuvants. The study highlighted that all the adjuvants enhanced the induction of systemic IgG. However, only CL413 could stimulate potent nasal IgA responses. Importantly, mice immunized with the dual pattern recognition receptor agonists adjuvanted-RSV-VLPs were protected against lung pathology after virus challenge while mice immunized with FI-RSV developed severe lung inflammation.

While most studies with RSV vaccines have focused on the use of adjuvants targeting a single PRR, many studies have shown that simultaneous targeting with two PRRs (such as TLRs and/or NLRs) can synergistically enhance the immune response (21, 22, 55, 56). However, a disadvantage of using two separate adjuvants is that they may not necessarily bind to the PRRs on or within the same cell. Our selection of the adjuvants was guided by this rationale that chimeric adjuvants will bind to two PRRs on or within the same cell and enhance antigen presentation. CL413 targets both TLR2 and TLR7, while CL429 can stimulate both TLR2 and NOD2 (57). For comparison, adjuvant combinations of Pam3CSK4+IMQ and Pam3CSK4+L18-MDP were used alongside the dual-PRR binding chimeric adjuvants.

The results demonstrated that CL413, but not Pam3CSK4+IMQ induced nasal IgA after pulmonary delivery and systemic humoral response (53). In line with our results, when the MPL (TLR4 ligand) and R837 (TLR7 ligand) were delivered in separate nanoparticles, the resulting immune response was lower than that induced by the nanoparticle incorporating both the ligands (23). The study was performed using influenza HA. Additionally, TLR2 and TLR7 ligands have been reported to have good mucosal immunoadjuvant properties (58, 59). It is noteworthy that in our study, IgA antibodies in the nasal cavity were induced against both preF and G proteins. PreF-specific IgA across both upper and lower respiratory compartments has been associated with protective immunity in studies in rodents and non-human primates (38, 60–62). Similarly, the delivery of an influenza vaccine candidate adjuvanted with a combined TLR2/TLR7 adjuvant elicited higher immune responses than unadjuvanted formulations (63). These findings highlight the importance of CL413 as a mucosal adjuvant in eliciting nasal IgA responses.

Earlier studies on mucosal immunity indicated that the microenvironment in the respiratory tract of mice favors Th2-dominated immune responses characterized by cytokines such as IL-4 and IL5 which drive the induction of Th2-associated antibody subtype IgG1 (64). In the present study, adjuvantation of the VLPs with CL413 overcame the Th2 microenvironment in the lungs and induced balanced IgG2a/IgG1 or skewed IgG2a response which is the outcome of the Th1 response. These results are in line with previous studies where the use of adjuvants have been shown to skew the immune response towards a favorable Th1 response (18, 61). While previous vaccine candidates incorporating a single TLR2 (61), TLR7 (62) or NOD2 (22) adjuvants have been investigated to demonstrate a Th1 polarized immune response; studies employing a dual TLR2/7 adjuvant and mucosal delivery have not been reported earlier to the best of our knowledge. CL413 is composed of Pam2CSK4 (a TLR2 ligand) conjugated to 8-hydroxyadenine (TLR7 agonist). Activation of these TLRs predominantly leads to the production of type I interferons and, consequently, a Th1 skewed adaptive response (65, 66).

To our surprise, the adjuvant CL429, induced skewed response towards IgG1. This outcome may be attributed to the intrinsic property of the CL429 adjuvant, which contains murabutide (a NOD2 ligand) covalently linked to Pam2C. It has been demonstrated that the activation of NLRs such as NOD1, NOD2, and NLRP3, results in the generation of a predominantly Th2 type of immune response (67). However, no pathology after RSV challenge was seen in the lungs of mice immunized with CL429 and other formulations that showed inferior IgG2a induction as compared to i.m. delivery. These results are in line with a study where DS-Cav-1 was administered with various adjuvants and RSV neutralization was achieved irrespective of an IgG1 or IgG2a predominant response (68). However, the failure to induce even a minimal Th1 response after immunization has been shown to mediate lung pathology (17). Overall, our experiments on antibodies show that serum IgG response is compensated by the IgA response in the lungs after pulmonary delivery and the IgG2a/IgG1 ratio is not as critical if sufficient IgG2a is available.

Consistent with the observed IgA antibody response, immunization with chimeric adjuvants resulted in an increased percentage of germinal center formation and proliferating B cells in the lungs of the mice. These findings suggest that pulmonary delivery of adjuvanted VLPs facilitates a robust mucosal B-cell response. It has been shown that B cells in the lung participate in protective humoral immune responses to RSV in humans (69). The enhanced B cells in the lungs might be due to the increased local secretion of IL-6 and IL-2 in the lung lymphocytes after *in vitro* stimulation as observed in our study (70, 71). Lung resident B cells, but not systemic memory B cells, have been shown to contribute to early plasmablast responses following challenge and in the control of respiratory viruses (43).

Cytokine profiling highlighted that the pulmonary immunization promoted almost all evaluated proinflammatory cytokines in the lungs. Interestingly, levels of anti-inflammatory IL-4 in the lungs that are shown to be associated with pathology of RSV in the airways were many folds lower compared to FI-RSV immunized mice. Although this study does not evaluate IL-13 and mucus production after mucosal delivery of chimeric adjuvants, it can be hypothesized that chimeric adjuvant might not induce IL-13, another important Th2 cytokine, that is shown to promote mucus production after RSV infection (72, 73). Inhibition of Th2 cytokines can be attributed to induction of IFN-γ or other pro-inflammatory cytokines induced by VLPs that are shown to inhibit the Th2 response and therefore IL-4 secretion from the cells. A special attention needs to be given to IL17-A induced by CL413 as its secretion is previously linked to IgA induction which is found in our study also. In the study conducted by Lindell et al. (2011), IL-17A induced locally in the lungs and BAL by a nano emulsion-based inactivated RSV immunization improved protection against RSV without increasing lung pathology or eosinophilia upon challenge in mouse and cotton rat models (74). Along with IFN-γ, IL-6 and IL-2, there was an increased secretion of TNF with the use of CL413 with the VLPs. TNF plays a critical role in the control of viral infection - through the recruitment and activation of macrophages, natural killer cells, T cells, and antigen-presenting cells (75). In a previous report by Su et al. (2008), IL-6 and TNF have been found to contribute a high IgG2a/IgG1 ratio and high expression level of IFN-γ in BALB/c mice when used as molecular adjuvants in a DNA vaccine, which agrees with the observations in the CL413 group in our study (76).

The quality and durability of neutralizing antibody responses and B cell memory depend tightly upon CD4+ T cell interaction. Moreover, CD4+ T cells are involved in a wide spectrum of activities critical to antiviral immunity (77). We found enhanced numbers of CD4+ T cells (total and resident) in mice immunized with adjuvanted formulations. Similarly, it was shown that pulmonary RSV-specific CD8+ T cells important for RSV clearance in the acute and late phases of infection (78). Induction of lung resident and proliferating CD4+ and CD8+ T cells is particularly important as the resident T cells are shown to proliferate exponentially upon antigen reencounter in lungs and contribute to timely protection. Consistent with our findings, essential roles of both local and peripheral T cells induced by vaccination in protection against RSV is documented (79). Overall, these observations of highlight the potential of use of adjuvants in enhancing the vaccine induced immune CD4+ and CD8+ T cell response (80).

The use of BALB/c and not cotton rats, the accepted animal model for RSV infection, is a limitation of our study. Lack of viral load estimation in the lungs of infected and challenged and protected mice is another limitation and restricts the depth of our analysis. However, the immune response shown in BALB/c mice is universally acceptable. We could not use cotton rats because of the issues with procurement and actual experimentation at our facility. Nevertheless, our findings align with numerous studies that have shown mice immunized with FI-RSV developing severe lung pathology when exposed to RSV, whereas protection has been achieved after immunization with antigens like BPL-inactivated RSV, low-energy electron inactivated RSV or other various forms of RSV-F proteins in unadjuvanted or adjuvanted formulations (79, 81–83).

In conclusion, of the chimeric adjuvants CL413 and CL429, CL413 was shown to be a promising mucosal adjuvant that can be used with RSV-VLPs. It could enhance nasal IgA, cytokine response in the lungs and modulate systemic response. CL429, on the other hand, though modulated cellular response to a limited extent in the lungs and spleen, led to limited systemic humoral responses. Of significance, pulmonary delivery of adjuvanted-VLPs modulated immune responses in the lungs where it matters the most during RSV infection. The chimeric adjuvants performed equally well as the mixture of adjuvants and even excelled in most aspects of the immune response. Thus, chimeric dual pattern recognition receptor ligands, especially CL413, are promising adjuvants for use in pulmonary vaccines against RSV.

## Data Availability

The raw data supporting the conclusions of this article will be made available by the authors, without undue reservation.

## Supplementary material

Supplementary Figure 1 Gating strategy for the analysis of B cell, CD4+ T cell and CD8+ T cell response after immunization.
