# Supplementary figures and images for: Evaluation of dual pathogen recognition receptor agonists as adjuvants for respiratory syncytial virus - virus-like particles for pulmonary delivery
Source: Front Immunol. 2025 Mar 17;16:1561297. doi: 10.3389/fimmu.2025.1561297 (PMC11962540; doi:10.3389/fimmu.2025.1561297)

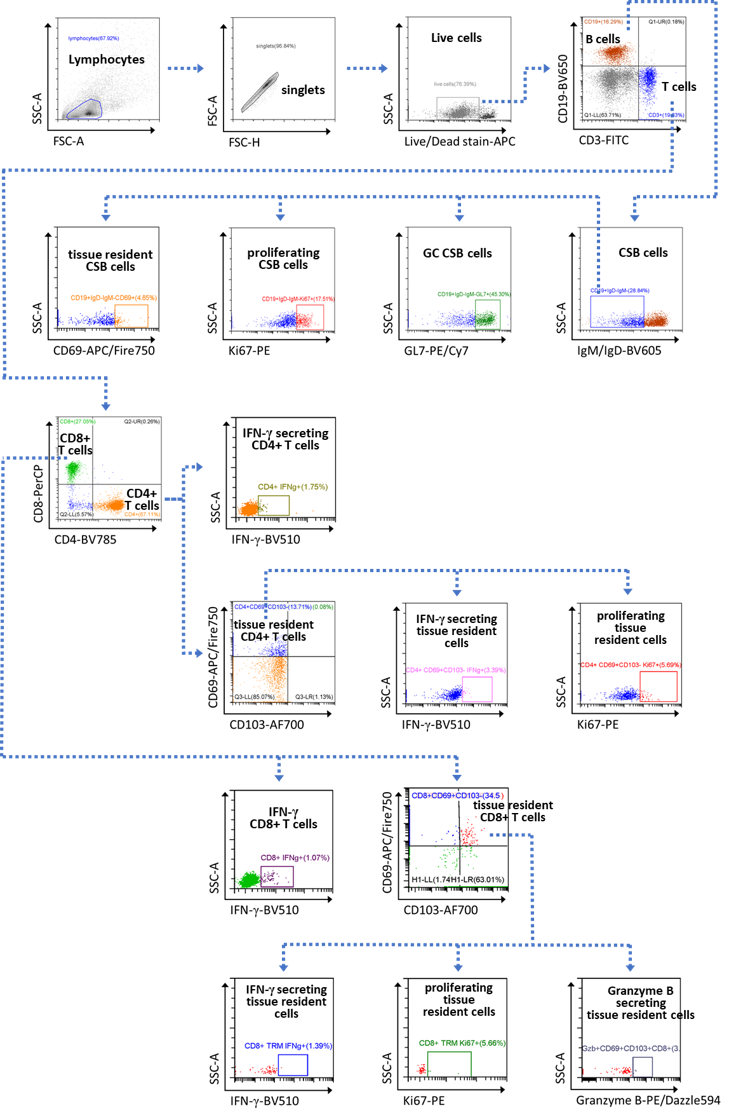

Supplement: Supplementary Figure 1 — Gating strategy for the analysis of B cell, CD4+ T cell and CD8+ T cell response after immunization. [file Image1.tif]
